# Genotype determination of the *OPN1LW*/*OPN1MW* genes: novel disease-causing mechanisms in Japanese patients with blue cone monochromacy

**DOI:** 10.1038/s41598-018-29891-9

**Published:** 2018-07-31

**Authors:** Satoshi Katagiri, Maki Iwasa, Takaaki Hayashi, Katsuhiro Hosono, Takahiro Yamashita, Kazuki Kuniyoshi, Shinji Ueno, Mineo Kondo, Hisao Ueyama, Hisakazu Ogita, Yoshinori Shichida, Hidehito Inagaki, Hiroki Kurahashi, Hiroyuki Kondo, Masahito Ohji, Yoshihiro Hotta, Tadashi Nakano

**Affiliations:** 1 Department of Ophthalmology, The Jikei University School of Medicine, Tokyo, Japan; 2 Department of Ophthalmology, Shiga University of Medical Science, Shiga, Japan; 3 Department of Ophthalmology, Katsushika Medical Center, The Jikei University School of Medicine, Tokyo, Japan; 4 Department of Ophthalmology, Hamamatsu University School of Medicine, Shizuoka, Japan; 5 Department of Biophysics, Graduate School of Science, Kyoto University, Kyoto, Japan; 6 Department of Ophthalmology, Kindai University Faculty of Medicine, Osaka, Japan; 7 Department of Ophthalmology, Nagoya University Graduate School of Medicine, Aichi, Japan; 8 Department of Ophthalmology, Mie University Graduate School of Medicine, Mie, Japan; 9 Department of Biochemistry and Molecular Biology, Shiga University of Medical Science, Shiga, Japan; 10 Division of Molecular Genetics, Institute for Comprehensive Medical Science, Fujita Health University, Aichi, Japan; 11 Department of Ophthalmology, University of Occupational and Environmental Health, Fukuoka, Japan

## Abstract

Blue cone monochromacy (BCM) is characterized by loss of function of both *OPN1LW* (the first) and *OPN1MW* (the downstream) genes on the X chromosome. The purpose of this study was to investigate the first and downstream genes in the *OPN1LW*/*OPN1MW* array in four unrelated Japanese males with BCM. In Case 1, only one gene was present. Abnormalities were found in the promoter, which had a mixed unique profile of first and downstream gene promoters and a −71A > C substitution. As the promoter was active in the reporter assay, the cause of BCM remains unclear. In Case 2, the same novel mutation, M273K, was present in exon 5 of both genes in a two-gene array. The mutant pigments showed no absorbance at any of the wavelengths tested, suggesting that the mutation causes pigment dysfunction. Case 3 had a large deletion including the locus control region and entire first gene. Case 4 also had a large deletion involving exons 2–6 of the first gene. As an intact LCR was present upstream and one apparently normal downstream gene was present, BCM in Case 4 was not ascribed solely to the deletion. The deletions in Cases 3 and 4 were considered to have been caused by non-homologous recombination.

## Introduction

The human retina contains three types of cone photoreceptors: long-wavelength sensitive cones (L cones), medium-wavelength sensitive cones (M cones), and short-wavelength sensitive cones (S cones). These cone photoreceptors express respective visual pigments, L, M, and S opsins. Among these, the genes encoding L opsin (*OPN1LW*, OMIM; *300822) and M opsin (*OPN1MW*, OMIM; *300821) are present in tandem on the human X chromosome^1,2^, forming an L/M pigment gene array. In individuals with normal color vision, the first gene in the array is an L gene, and the downstream (the second and later) gene(s) is/are M gene(s). Abnormalities in the array are reportedly associated with protan and deutan color vision deficiencies^3^, blue cone monochromacy (BCM)^4^, and Bornholm eye disease^5^.

BCM (OMIM; #303700) is a rare congenital color vision deficiency with an X-linked inheritance pattern^4,6^. Cases of BCM typically present with severely impaired color discrimination, reduced visual acuity, nystagmus, photophobia, and diminished L/M cone function despite retention of rod and blue cone function^6,7^. The dysfunction in both L and M cones in BCM is reportedly caused by one of the three genotypes. One genotype involves deletion of the locus control region (LCR)^4,8–12^, which is located upstream of the L/M gene array (−3,681 to −3,021 from the cap site of the first gene) and believed to be involved in the mutually exclusive expression of L and M genes^13,14^. Therefore, neither gene is expressed in the absence of the LCR. Another genotype involves a deleterious mutation in a single-gene array (either the L or M gene present alone in the array). The derivation of this genotype has two obvious steps: first, non-homologous recombination between the L and M genes to form a single-gene array followed by an inactivating mutation in the single gene (reverse order is also possible). The most common mutation is C203R^4,8,15–17^, but other mutations, such as P307L^8^, R247X^8^, and deletion of exon 2^16^, have also been documented. The LIAVA haplotype in exon 3, which affects splicing^18^, was also reported in a single L gene^17^. The third genotype involves inactivating mutations in both the L and M genes. Although the C203R mutation has been documented in this genotype^8,16,19^, the LIAVA haplotype (or a very similar haplotype) in exon 3 of both genes seems to be frequent^17,20^.

Although little is known about the prevalence of BCM in the Japanese population, to date, only two BCM families have been described in the literature, demonstrating the mechanism of deletion of the LCR in both families^11,21^.

In the current study, the L/M pigment gene arrays in four unrelated Japanese males with BCM were analyzed. The purpose of this study was to investigate their genotypes in the L/M pigment gene array, which could be categorized into one of the three above-mentioned genotypes, but others were unreported mechanisms and differed from each other.

## Results

### Case 1 (JU#1299)

**Figure 1:**
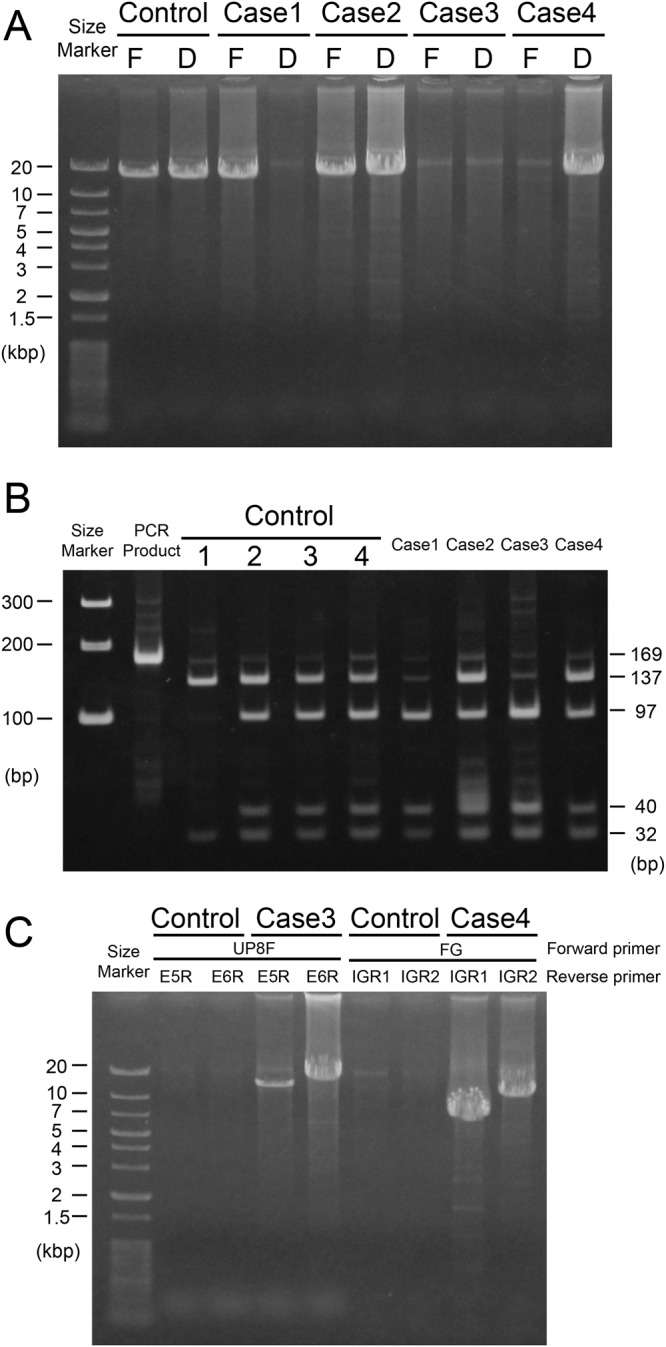
Long-range PCR and promoter analysis. (**A**) First and downstream genes in the L/M gene array were amplified separately by long-range PCR. The control was a color-normal subject having both the first and downstream genes. F, first gene; D, downstream gene(s). Thin bands of approximately 20 kb are not amplified products but the templates (genomic DNA, usually approximately 100 ng per reaction). (**B**) Promoter analysis of gene number. Promoters were amplified by PCR using primers common to the first and downstream genes. PCR products (169 bp) digested with *Cfr*10I were loaded onto a polyacrylamide gel. Controls 1–4 have gene numbers 1–4, respectively^28^. (**C**) Long-range PCR beyond the deletion. Combinations of the primers UP8F/E5R and UP8F/E6R were used for long-range PCR in the control and Case 3. Combinations of the primers FG/IGR1 and FG/IGR2 were used for long-range PCR in the control and Case 4.

**Figure 2:**
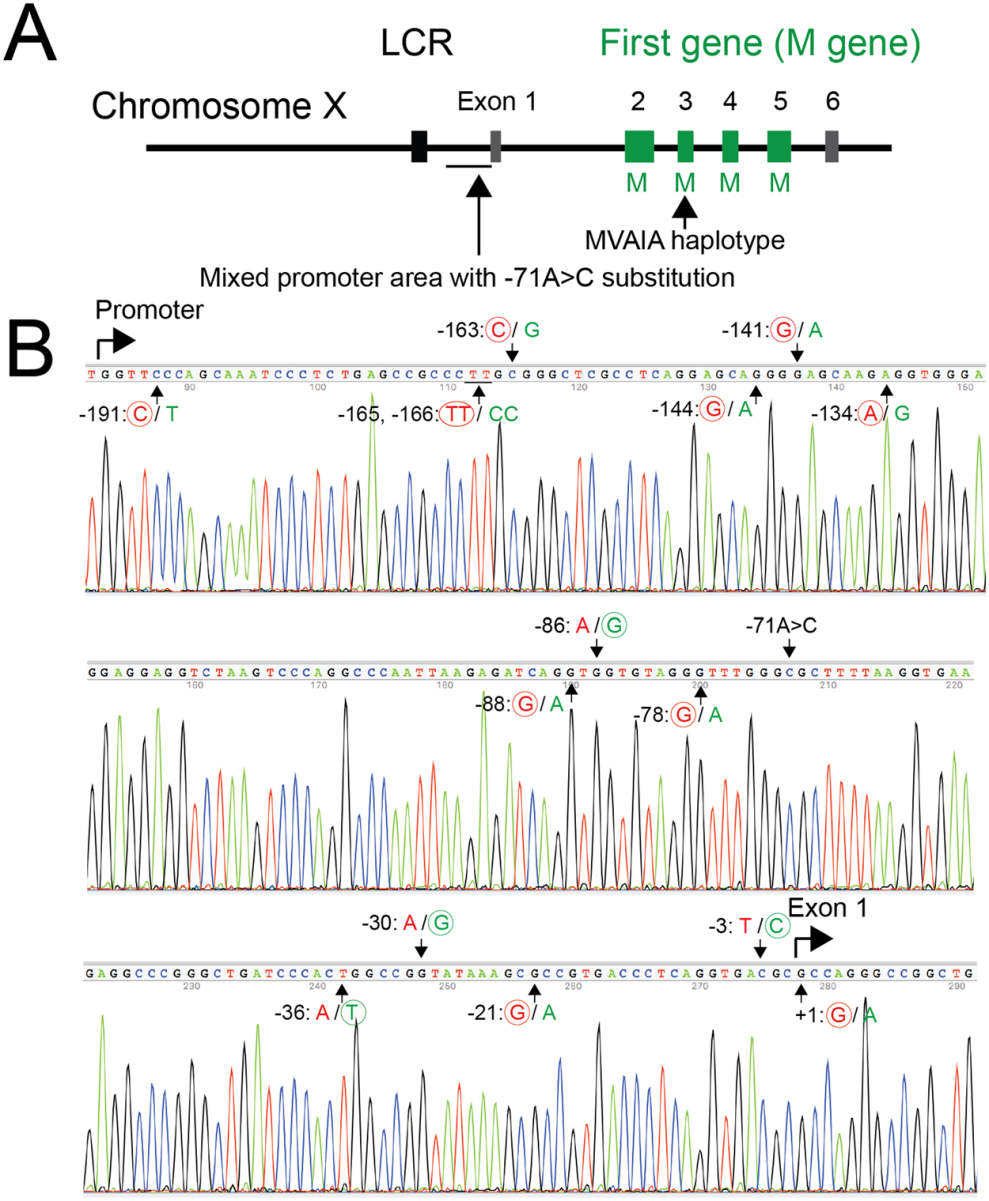
Genotype of Case 1. (**A**) Overview of the genotype of Case 1. Case 1 had an intact LCR and a single M gene array in which no aberrations were found. The promoter regions had a unique profile including a −71 A > C substitution. (**B**) The promoter of the single M-gene array. Black arrows indicate the 14 nucleotides differing between first and downstream genes and the −71 A > C substitution. At each position, the usual nucleotide of the first gene promoter is shown on the left side in red and that of the downstream gene promoter is shown on the right side in green. The nucleotides in Case 1 are circled.

Long-range polymerase chain reaction (PCR) was successful for the first gene but not for downstream gene(s) (Fig. 1A). Promoter analysis of gene number, by contrast, showed that the first gene promoter was absent (only the downstream gene promoter was detected) (Fig. 1B). From the results of repeated long-range PCR analysis of downstream genes, we concluded that this subject had a single gene (no downstream genes) in the array. The LCR was present upstream of the single gene, and its sequence had no aberrations. The curious result that this subject had only the downstream gene promoter (Fig. 1B) was later found to be due to the unique sequence of the promoter. The first and downstream gene promoters differ by 14 nucleotides, but in the promoter region of the single gene, 8 upstream sites were associated with the first gene, whereas the other 6 sites were a random mixture of these nucleotides (Fig. 2). Moreover, the promoter had a −71 A > C substitution, which has been reported to be associated with deutan color vision deficiency^22^ (Fig. 2). The G at the −30 position in the ^−34^GCCGGT^−29^ sequence (the number is from the cap site of the first gene) in the promoter analysis indicated that the first gene promoter was absent (Fig. 1B). The −30 (A or G) site discriminates the promoters of the first and downstream genes by *Cfr*10I (recognition sequence = RCCGGY). Our conclusion was that Case 1 had a single gene array because the sequencing of the promoter showed only one curious pattern as shown in Fig. 2. The luciferase activity of the promoter of the single gene was more than twice that of the usual first gene promoter in the reporter assay (Supplementary Fig. S1). No abnormalities were found in exons 1–6 and their adjacent introns in nucleotide sequencing. Exons 2–5 were M type, and the haplotype of exon 3 was MVAIA rather than LIAVA (Table 1). The curious promoter found in Case 1 has not been reported previously.

**Table 1:** Nucleotides in each gene of the four cases.

		Exon 2				Exon 3								Exon 4					Exon 5											Haplotype in Exon 3*
Reference	Nucleotide position	194	300	331	347	453	457	465	511	513	521	532	538	689	697	698	699	706	820	823	825	828	830	835	849	853	888	892	926	
	L gene	C	A	A	C	G	C	G	G/A	G/T	C/T	A/G	T	T	G	C	T	A	A	T	T	G	A	G	C	A	T	G	A	
	M gene	T	G	G	A	A	A	C					G	C	A	G	C	G	G	C	G	A	T	T	A	G	C	C	T	
	Amino acid Position	65	100	111	116	151	153	155	171–1	171–3	174	178	180	230	233–1	233–2	233–3	236	274	275–1	275–3	276	277	279	283	285	296	298	309	
	L gene	T	L	I	S	R	L	V	V/I		A/V	I/V	S	I	A			M	I	F		A	Y	V	P	T	G	A	Y	
	M gene	I	L	V	Y	R	M	V					A	T	S			V	V	L		A	F	F	P	A	G	P	F	
Case 1 First (single) gene	Nucleotide	T	G	G	A	A	A	C	G	G	C	A	G	C	A	G	C	G	G	C	G	A	T	T	A	G	C	C	T	MVAIA
	Amino acid	I	L	V	Y	R	M	V	V		A	I	A	T	S			V	V	L		A	F	F	P	A	G	P	F	
Case 2 First gene	Nucleotide	C	A	A	C	G	C	G	G	G	C	A	T	T	G	C	T	A	G	C	G	A	T	T	A	G	C	C	T	LVAIS
	Amino acid	T	L	I	S	R	L	V	V		A	I	S	I	A			M	V	L		A	F	F	P	A	G	P	F	
Case 2 Downstream gene	Nucleotide	T	G	G	A	A	A	C	G	G	C	A	G	C	A	G	C	G	G	C	G	A	T	T	A	G	C	C	T	MVAIA
	Amino acid	I	L	V	Y	R	M	V	V		A	I	A	T	S			V	V	L		A	F	F	P	A	G	P	F	
Case 3 Downstream gene (First gene was deleted)	Nucleotide	C	A	A	C	A	A	C	G	G	C	A	G	T	G	C	T	A	A	T	T	G	A	G	C	A	T	G	A	MVAIA
	Amino acid	T	L	I	S	R	M	V	V		A	I	A	I	A			M	I	F		A	Y	V	P	T	G	A	Y	
Case 4 Downstream gene (First gene was deleted)	Nucleotide	T	G	G	A	G	C	G	G	G	T	G	G	C	A	G	C	G	G	C	G	A	T	T	A	G	C	C	T	LVVVA
	Amino acid	I	L	V	Y	R	L	V	V		V	V	A	T	S			V	V	L		A	F	F	P	A	G	P	F	

The positions of nucleotides different between wild-type L and M genes, and polymorphic nucleotide positions 511, 513, 522 and 532 as well, are shown. *Haplotype in Exon 3 was determined by amino acid residues at 153, 171, 174, 178, and 180.

### Case 2 (JU#1311, KINKI-125-70)

**Figure 3:**
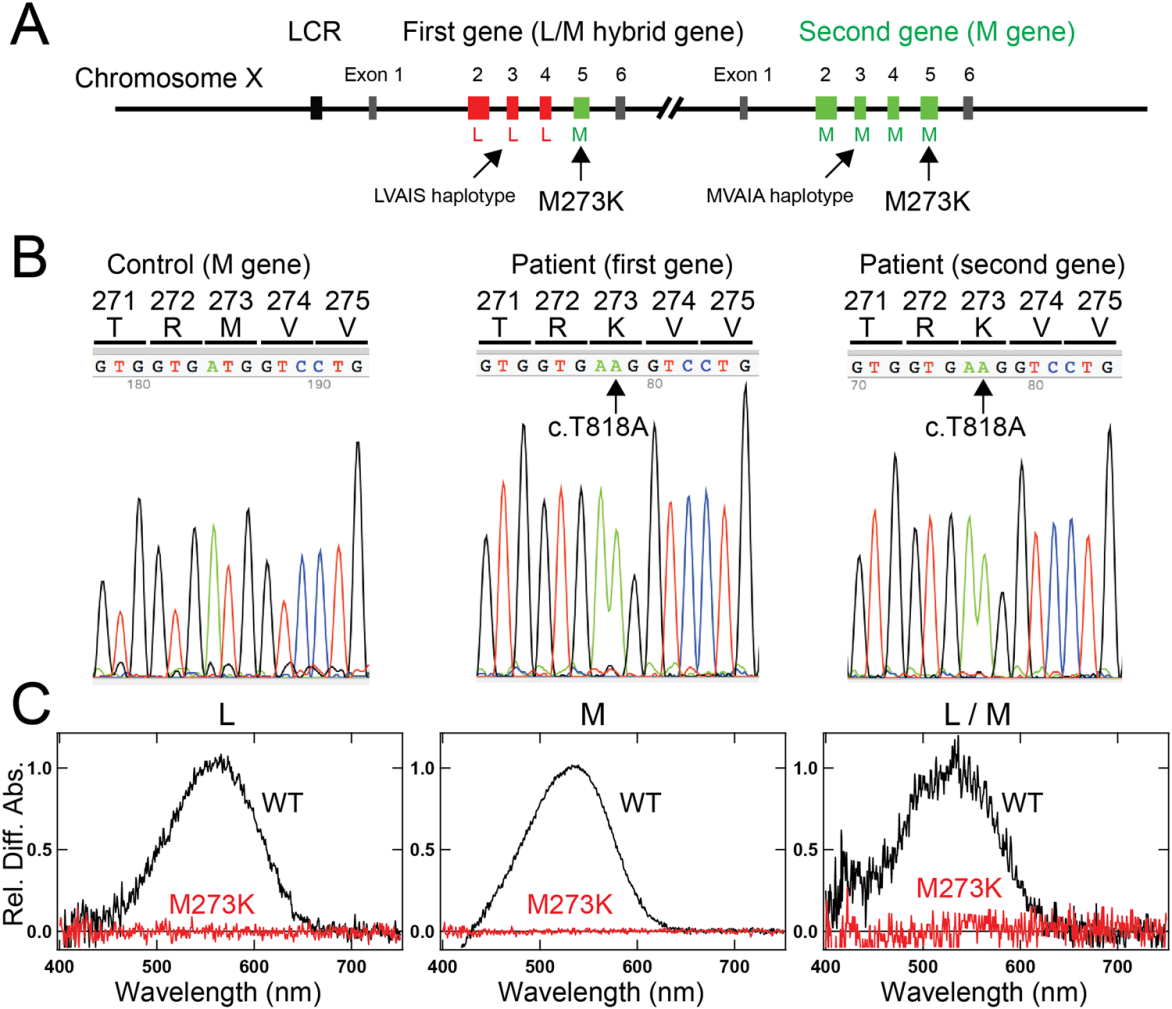
Genotype of Case 2. (**A**) Overview of the genotype of Case 2. Case 2 had an intact LCR and two genes. The first gene had exons 2–4 of L type, exon 3 with LVAIS haplotype, and exon 5 of M type. The second gene had exons 2–5 of M type and exon 3 with MVAIA haplotype. Both genes had the same missense mutation (c.818 T > A, M273K) in exon 5. (**B**) Partial sequence data around the missense mutation (c.818 T > A, M273K) in exon 5 in the control and two genes of Case 2. (**C**) Opsin reconstitution experiments. L, L opsin in which exons 2–5–derived amino acid sequences are all L type; M, M opsin in which exons 2–5–derived amino acid sequences are all M type, as in the product of the second gene of Case 2; L/M, M opsin in which exons 2–4–derived amino acid sequences are L type but exon 5–derived amino acid sequence is M type, as in the product of the first gene of Case 2. WT, wild-type opsin; M273K, mutant opsin with the M273K mutation. “Rel. Diff. Abs.” indicates relative difference absorption.

Products of both the first and downstream genes were obtained using long-range PCR (Fig. 1A). Promoter analysis showed that the subject had a 2-gene array (Fig. 1B). Both genes had the same missense mutation (c.818 T > A, M273K) in exon 5 (Fig. 3A,B). The chromosome positions (GRCh38.p7) of the mutation are 154,156,367 (L gene) and 154,193,481 (M gene). The M273K mutation has not been reported previously, and not found in the Single Nucleotide Polymorphism Database (https://www.ncbi.nlm.nih.gov/projects/SNP/), Genome Aggregation Database (http://gnomad.broadinstitute.org/), Exome Aggregation Consortium (http://exac.broadinstitute.org/) and Human Genetic Variation Database (http://www.hgvd.genome.med.kyoto-u.ac.jp/). The analysis of the recombinant proteins of M273K mutants revealed that the opsin with the M273K mutation was significantly detectable in the Western blot and cultured cells (Supplementary Fig. S2) but showed no absorbance at any of the wavelengths tested after reconstitution with 11-*cis*-retinal (Fig. 3C). These results indicated that the M273K mutation in both genes results in dysfunctional opsin protein, probably because of a lack of the ability to bind to 11-*cis*-retinal. We therefore ascribed the BCM phenotype in this subject to the mutation. In the first gene, exons 2, 3, and 4 were L type, exon 5 was M type, and the haplotype of exon 3 was LVAIS. In the downstream gene, exons 2–5 were M type, and the haplotype of exon 3 was MVAIA (Table 1).

### Case 3 (JU#1318, MIE-050-0071)

**Figure 4:**
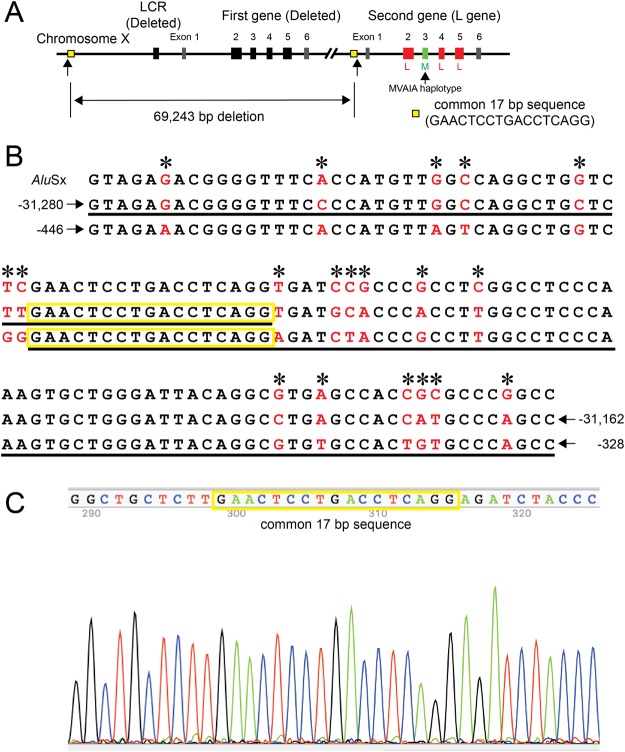
Genotype of Case 3. (**A**) Overview of the genotype of Case 3. Case 3 had a large deletion of 69,243 bp including the LCR and first gene. The remaining second gene was an L gene in which exons 2, 4, and 5 were L type. Exon 3 was M type with MVAIA haplotype. (**B**) Upper row shows the consensus sequence for the left monomer of the *Alu* element (complementary sequence of AluSx). Middle row shows a part of the upstream region of the first gene (number is from the cap site of the first gene). Lower row shows a part of the upstream region of the downstream gene (number is from the cap site of the downstream gene). The nucleotides differing among the regions are shown in red and by asterisks. The actual sequence obtained in Case 3 is underlined. (**C**) Partial sequence data around the breakpoint of the deletion. The breakpoint is somewhere in the common 17-bp sequence.

Long-range PCR was unsuccessful for both the first and downstream genes (Fig. 1A). Promoter analysis for determining gene number showed that the first gene promoter was absent (only the downstream gene promoter was detected) (Fig. 1B). Amplification of the LCR was also unsuccessful, indicating a deletion including both the LCR and first gene. To determine the exact deletion breakpoints, 11 sets of PCR primers were designed to cover the sequence −53,930 to −9,320 (number is from the cap site of the first gene) (NT_025965.12: 707,760 to 752,370). PCR products were obtained when using the UP8F and UP8R pair (Supplementary Table S1) but not the UP9F and UP9R pair (Supplementary Table S1), suggesting that the upstream breakpoint of the deletion was between −32,015 and −28,150 (NT_025965.12: 729,665 to 733,530). Long-range PCR using the primer sets UP8F/E5R and UP8F/E6R was successful in this subject but not in a color-normal control subject (Fig. 1C). According to the human genome database (NT_025965.12), the distance between UP8F- and E5R-corresponding regions and UP8F- and E6R-corresponding regions were 81,131 bp and 83,925 bp, respectively (the E5R- and E6R-corresponding regions are those of the downstream gene), which were too far for long-range PCR. In Case 3, however, due to the large deletion including the first gene, the distances between the regions had been reduced to about 12 kbp and 15 kbp, respectively, and therefore, long-range PCR products were obtained in this case (Fig. 1C). The 15-kbp product contained not only exons 1–6 (exons 2, 4, and 5 were L type, exon 3 was M type, with the haplotype of MVAIA) (Table 1) but also the downstream gene promoter. By sequencing the 15-kbp product using the UP12F primer (Supplementary Table S1), the upstream breakpoint of the deletion was determined to be somewhere in the sequence ^−31,241^GAACTCCTGACCTCAGG^−31,225^ (the number is from the cap site of the first gene) (NT_025965.12: 730,439 to 730,455), and the downstream breakpoint was determined to be somewhere in the sequence ^−407^GAACTCCTGACCTCAGG^−391^ (the number is from the cap site of the downstream gene) (NT_025965.12: 799,682 to 799,698) (Fig. 4). The reason why long-range PCR was unsuccessful for the downstream gene (Fig. 1A) is that the deletion includes the region corresponding to the forward primer for long-range PCR, DG (−748 to −728 from the cap site of the downstream gene). The estimated size of the deletion was 69,243 bp. The long-range PCR products in Fig. 1C were calculated to be exactly 11,888 bp and 14,682 bp. As a LCR is reportedly necessary for the expression of L/M genes^13,14^, the BCM phenotype in this subject was ascribed to the deletion.

### Case 4 (JU#1368, Nagoya-140)

Long-range PCR was successful for downstream gene(s) but not for the first gene (Fig. 1A). Promoter analysis of gene number showed a 1:1 ratio for the first and downstream genes (Fig. 1B). As the first gene promoter was shown to be present, the FG primer–corresponding region (upstream of the promoter) should also be present. PCR analysis using combinations of the FG primer and various intragenic reverse primers revealed that the upstream breakpoint of the deletion was within intron 1 (between the primer I1R1– and primer I1R2–corresponding regions) and that the deletion expanded beyond exon 6. The failure of long-range PCR for the first gene was ascribed to the absence of exon 6 (primer E6R corresponds to exon 6). To determine the downstream breakpoint of the deletion, 15 reverse primers specific to the intergenic region (between the first and downstream genes) were designed for long-range PCR. PCR products were obtained for two primer pairs (FG/IGR1, and FG/IGR2) in this subject but not in the control (Fig. 1C). According to the human genome database (NT_025965.12), the distances between the FG- and IGR1-corresponding regions and between the FG- and IGR2-corresponding regions were 30,137 bp and 34,286 bp, respectively, which were too long for long-range PCR. In Case 4, however, due to the large deletion, the distances had been reduced to approximately 7 kbp and 11 kbp, respectively, and therefore, long-range PCR products were obtained (Fig. 1C).

**Figure 5:**
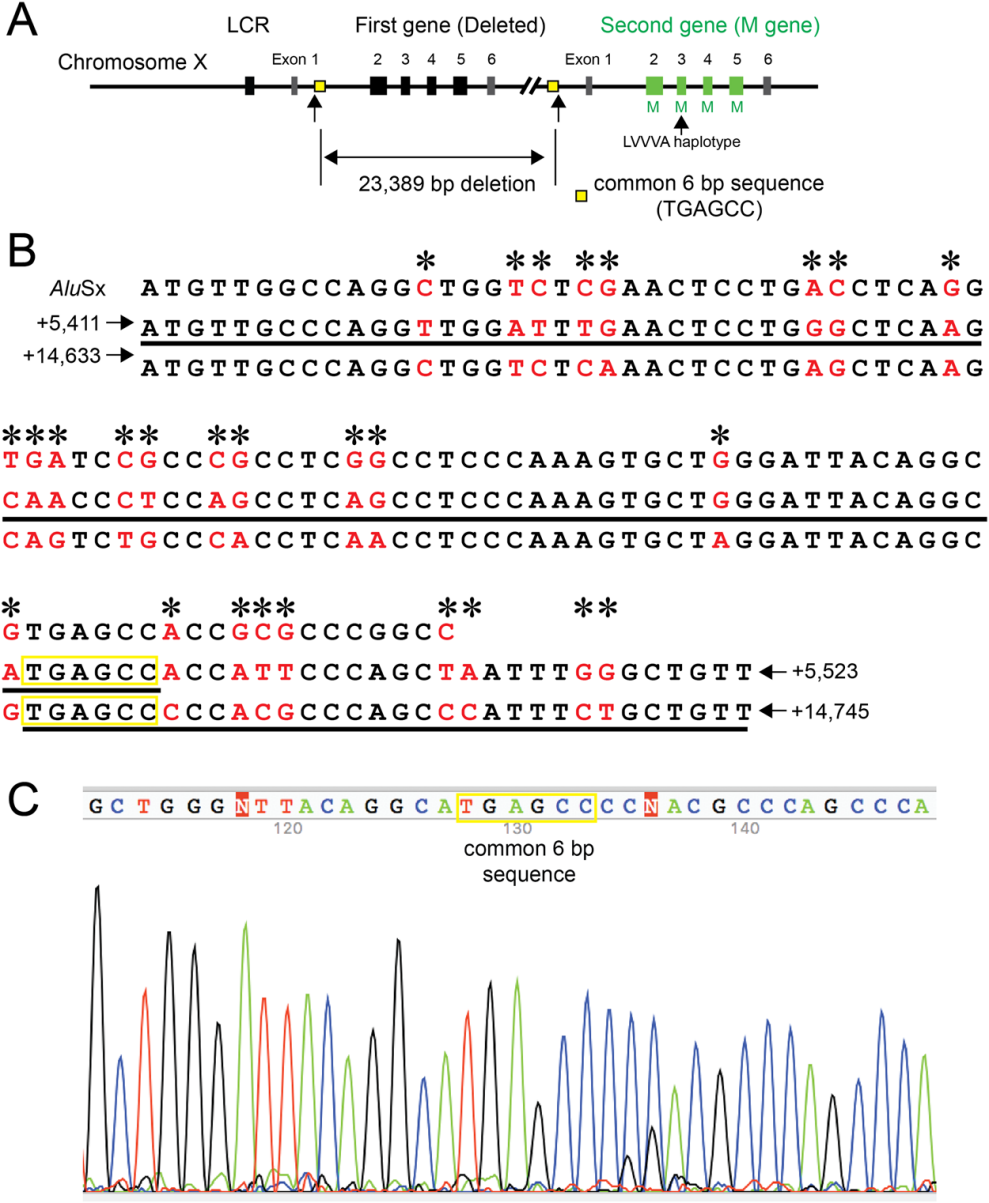
Genotype of Case 4. (**A**) Overview of the genotype of Case 4. Case 4 had a large deletion of 23,389 bp including exons 2–6 of the first gene. The intact LCR and second gene were present. The second gene had exons 2–5 of M type and exon 3 with LVVVA haplotype. (**B**) Upper row shows the consensus sequence for the left monomer of the *Alu* element (complementary sequence of AluSx). Middle row shows a part of intron 1 of the first gene (the number is from the 5′ splice site of intron 1). Lower row shows a part of the intergenic region (the number is from the stop codon in exon 6 of the first gene). The nucleotides differing among the regions are shown in red and by asterisks. The actual sequence obtained in Case 4 is underlined. (**C**) Partial sequence data around the breakpoint of the deletion. The breakpoint is somewhere in the common 6-bp sequence.

The IGR1 primer corresponds to the region +14,867 to +14,887 (the number is from the stop codon in exon 6) (NT_025965.12: 791,231 to 791,251), and the IGR2 primer corresponds to the region +15,232 to +15,252 (NT_025965.12: 767,324 to 767,329). Using the 11-kbp PCR product and primer I1F (Supplementary Table S1), the upstream breakpoint of the deletion was determined to be somewhere in ^+5,492^TGAGCC^+5,497^ (the number is from 5′ splice site of intron 1 of the first gene) (NT_025965.12: 767,324 to 767,329), and the downstream breakpoint was determined to be somewhere in ^+14,714^TGAGCC^+14,719^ (the number is from the stop codon in exon 6) (NT_025965.12: 790,713 to 790,718) (Fig. 5). The estimated size of the deletion was 23,389 bp. The long-range PCR products shown in Fig. 1C were calculated to be exactly 6,748 bp and 10,897 bp. PCR confirmed the presence of the LCR, and its sequence had no aberrations. The downstream gene had no abnormalities in the promoter, exons 1–6, or their adjacent introns. Exons 2–5 were M type, and the haplotype of exon 3 was LVVVA (Table 1). As the downstream gene was apparently normal, the cause of BCM in this subject could not be confidently determined.

## Discussion

In this study, we reported the results of gene analyses in four cases of BCM. Their genotypes were unreported and different from each other.

Case 1 had a single-gene array having a curious promoter sequences with a −71 A > C substitution. The −71 A > C substitution was reported to be associated with deutan color vision deficiency due to decreased promoter activity^22^. We hypothesized that the −71 A > C substitution causes dysfunction of the single gene. However, rather than being low, the activity of the promoter in Case 1 was more than twice that of the control in the reporter assay. It is reported that not only the LCR but also normal promoters in the L/M gene array were essential for expression of both L and M genes^13,14^. The LCR was not contained in the constructs for our reporter assay system. Although the dysfunction of the single opsin gene in Case 1 remains unclear, it might be possible that the curious promoter sequences (Fig. 2) might interfere with the LCR binding to the promoter.

Case 2 had a two-gene array, and both genes had a novel missense mutation (M273K) that would cause dysfunction of both gene products (Fig. 3C). The C203R mutation in exon 4 reportedly causes loss of function of the L/M gene products; this mutation causes deutan color vision deficiency when present in the M gene^23,24^ and BCM when present in both the L and M genes^8,16,19^. The occurrence of C203R mutation in both the L and M genes was explained by gene conversion^19^ (i.e., transfer of the C203R mutation present in the downstream M gene to the first L gene). The deleterious LIAVA haplotype in exon 3 of both the L and M genes was also explained by gene conversion^20^. Also, the occurrence of the M273K mutation in both the first and second genes (Fig. 3A) might be explained by the same mechanism of gene conversion seen in the C203R mutation^19^. Otherwise, because the first gene in this subject had L-type exons 2–4 and M-type exon 5 (with the mutation), we developed the following hypothesis as the other alternative possibility. The M273K mutation occurred in the downstream M gene (the array was L-M*; *denotes the mutation), duplication of the M gene occurred (L-M*-M*), followed by non-homologous recombination (L/M* hybrid-M*) as shown in Fig. 3A. Duplication of the second gene was supported by the fact that (i) many color-normal individuals have multiple downstream M genes with the same nucleotide sequence^25^ and (ii) the result that three (or more) downstream M genes had the same 11-bp deletion in a protanopia subject^26^. Case 2 had a genotype with two unique profiles; the M273K mutation was novel and present in both L and M genes.

Cases 3 and 4 showed large deletions of 62,934 bp including the LCR and 23,389 bp not including the LCR, respectively. The genotype of Case 3 was consistent with the known genotype of BCM. In Case 4, the first gene was obviously non-functional due to the absence of exons 2–6, but the downstream gene seemed to be functional, as no deleterious mutations were found in the promoter and exons, including their adjacent introns. The clinical phenotype of BCM in Case 4 indicated that the downstream gene was non-functional. The gene array revealed that the promoter of the second gene was directly connected to intron 1 of the first gene with absence of exons 2–6 (23,389 deletion) (Fig. 5). Although we cannot explain reasonable mechanisms underlying BCM in Case 4, the residual sequences (exon 1 and partial intron 1) of the first gene might impact on the promoter activity of the second gene, resulting in suppression of the second gene expression. The breakpoints of the deletion were within *Alu* elements in both cases, as in previously reported BCM cases^11^. Many reports have described deletions in the L/M gene array^4,8–12,17^. However, few studies have determined the exact breakpoints of the deletion at the nucleotide level^4,11,16^; the breakpoints have resulted from simple breakage and fusion^4,16^ outside the repetitive sequence and simple breakage and fusion (and insertion) in *Alu* elements^11^. The breakpoints we determined differed from these; non-equal crossing-over occurred between the two *Alu* elements in the region of the same sequence (Figs 4 and 5).

The various haplotypes of five amino acid residues at positions 153 (L/M), 171 (V/I), 174 (A/V), 178 (I/V), and 180 (S/A) in exon 3 have been reported in subjects with normal color vision and subjects with color vision deficiencies^18,20^. The haplotypes have been roughly classified into four groups in terms of the magnitude of the splicing defect^20^; highly deleterious haplotypes include LIAVA, MIAVA, and LVAVA; intermediately deleterious haplotypes include LIAIA, LIAVS, and MVAVA, minor deleterious haplotypes include LVAIA, LVAIS, MVAIA, and MVVVA, and the MVAIS haplotype exhibits no splicing defect. According to our data, the MVAIA and LVAIS haplotypes in Cases 1–3 would be expected to produce essentially correct splicing; however, the LVVVA haplotype in Case 4 could not be classified, as this haplotype was not described in the above-mentioned study^20^. We^18^ and other researchers^27^ examined the LVVVA haplotype using a mini-gene system and observed that the opsin mRNA retaining exon 3 was in clearly greater abundance than that lacking exon 3. Moreover, we reported one color-normal subject in which the exon 3 haplotype was LVVVA^18^. Based on these results, the BCM phenotype in Case 4 could not be ascribed to the LVVVA haplotype in the downstream gene.

In conclusion, we reported four novel and different genotypes in four unrelated Japanese patients with BCM. In two patients (Case 2 and Case 3), the genotypes were consistent with that of BCM (the same deleterious mutation in both opsin genes and deletion of the LCR), but in the other two cases (Case 1 and Case 4), the cause of BCM could not be clearly determined, although the patients exhibited very unique genotypes.

## Methods

The protocol for this study was approved by the Institutional Review Boards of The Jikei University of Medicine, Shiga University of Medical Science, Hamamatsu University School of Medicine, Kyoto University, Kindai University Faculty of Medicine, Nagoya University Graduate School of Medicine, Mie University Graduate School of Medicine, and Fujita Health University. The protocol adhered to the tenets of the Declaration of Helsinki, and informed consent was obtained from all participants.

### Participants

We recruited four unrelated Japanese male patients with BCM, whose diagnosis of BCM was made according to the findings reported^6,21^. In brief, all participants exhibited clinical findings of BCM, such as decreased visual acuity, severely impaired color discrimination in color vision tests, diminished L and M cone functions but retained S cone function in electroretinography, and an X-linked inheritance pattern in the family history. The detailed clinical findings are summarized in Supplementary Table S2.

### Molecular genetic analysis

Genomic DNA was extracted from leucocytes in venous blood samples using a Gentra Puregene blood kit (Qiagen, Hilden, Germany). First and downstream genes of the L/M visual pigment gene array were separately amplified by long-range PCR using a QIAGEN LongRange PCR kit (Qiagen). Primers FG and E6R were used for the first gene, and primers DG and E6R were used for downstream gene(s) (Supplementary Table S1). The position of primers used in this study are schematically shown in Supplementary Fig. S3. Primer E6R was common to both genes, but primers FG and DG were designed specifically for the upstream region of each gene. The cycling parameters were: 93 °C for 3 min; 10 cycles of 93 °C for 30 s, 62 °C for 30 s, and 68 °C for 15 min; then 18 cycles of 93 °C for 30 s, 62 °C for 30 s, and 68 °C for 15 min, with a 20-s increment per cycle. The resulting PCR products were used as templates for sequencing the ‘promoter + exon 1’ and exons 2–6, including their adjacent introns, using a BigDye Terminator v3.1 Cycle Sequencing kit (Thermo Fisher Scientific, Waltham, MA, USA) and ABI 3130*xl* sequencer (Thermo Fisher Scientific). The primer pairs used for sequencing are listed in Supplementary Table S1.

The LCR, which is located about 3.5 kb upstream of the first gene, was amplified by PCR using primers LCRF and LCRR, and its nucleotide sequence was then determined. When deletion was suspected, multiple sets of primers were designed for PCR to determine the exact deletion breakpoints.

Array gene number was determined by promoter analysis, as previously described^22^. Briefly, the promoters were amplified by PCR using Takara *Taq* DNA polymerase (Takara Bio Inc., Kusatu, Japan) and the primer pair common to both genes (Supplementary Table S1). The 169-bp PCR product was digested with *Cfr*10I (Takara) and analyzed by electrophoresis on a polyacrylamide gel. The first gene promoter was expected to yield two DNA fragments (137 bp and 32 bp), whereas the downstream gene promoter was expected to yield three DNA fragments (97 bp, 40 bp, and 32 bp). Gene number was estimated from the fluorescence ratio ([137 bp +97 bp]/137 bp). Gene Ladder Wide 2 was used as the size marker (Nippon Gene Co., Ltd., Toyama, Japan). Genomic DNAs from four subjects in which the L/M gene number was confirmed to be 1–4 by pulsed-field gel electrophoresis and Southern blotting^28^ were used as control templates.

### Promoter assay

To evaluate the activity of the promoter region, a luciferase reporter assay was performed as previously described^29^. Briefly, the promoter region of interest (−190 to +41 from the cap site of the gene) was amplified by PCR using Phusion High-Fidelity DNA polymerase (New England BioLabs, Ipswich, MA, USA) and restriction site–tagged primers (*Nhe*I site upstream and *Hin*dIII site downstream). The PCR product was cloned between the *Nhe*I and *Hin*dIII sites of a luciferase reporter plasmid, pGL4.17 (Promega Corp., Fitchburg, WI, USA). The resulting plasmid was transfected into WERI Rb1 cells using X-tremeGENE 9 DNA transfection reagent (Sigma-Aldrich, St. Louis, MO, USA). Two days after transfection, the cells were collected and lysed using PicaGene Cell Culture Lysis Reagent Luc (Wako Chemicals, Osaka, Japan). Luciferase activity was measured using a luminometer (Lumicounter NU-2500, Microtech Co., Ltd., Funahashi, Japan) and PicaGene Luminescence kit (Wako). Transfection efficiency was monitored in cells co-transfected with a ß-galactosidase–encoding plasmid (Promega).

### Analysis of M273K mutant pigments

The cDNAs of human L and M pigments and respective hybrid pigment were tagged with the epitope sequence of the anti-bovine rhodopsin monoclonal antibody Rho1D4 (ETSQVAPA) at the C terminus and were inserted into the mammalian expression vector pMT4^30^. cDNAs harboring the mutation M273K were constructed using an In-Fusion cloning kit (Takara). For the spectral analysis, the plasmid DNA was transfected into HEK293 cells using the calcium-phosphate method^31^. After 2 days of incubation, the cells were collected by centrifugation and supplemented with 11-*cis*-retinal in buffer A (50 mM Hepes [pH 6.5] and 140 mM NaCl) to reconstitute the pigments. The reconstituted pigments were extracted using 0.75% CHAPS and 1 mg/mL phosphatidylcholine in buffer A. Absorption spectra of the extracted pigments were recorded at 0 °C using a Shimadzu UV-2450 spectrophotometer. The pigments were irradiated with orange light through an O58 cutoff filter (Toshiba, Tokyo, Japan) for 1 min. Difference spectra were calculated from spectra recorded before and after irradiation. For the western blot analysis, extracts from pigment-transfected or mock-transfected HEK293 cells were subjected to SDS-PAGE, transferred onto a polyvinylidene difluoride membrane, and probed with Rho1D4. Immunoreactive proteins were detected by ECL Western Blotting Detection Reagents (GE Healthcare, United Kingdom) and visualized by a luminescent image analyzer (LAS 4000mini, GE Healthcare). For the fluorescence microscopy analysis, pigment-transfected or mock-transfected HEK293 cells were seeded onto poly-L-lysine coated coverslips. After 24 h incubation, cells were fixed in cooled methanol for 5 min. After fixation, cells were washed three times in PBS and were incubated overnight with primary antibody, Rho1D4, in 10% normal goat serum at room temperature. Cells were washed three times in PBS and were incubated for 1 h with secondary antibody, Alexa Fluor 488 anti-mouse IgG, in 10% normal goat serum at room temperature. Cells were washed a final time and were mounted onto slides with home-made aqueous mounting media consisting of glycerol and polyvinyl alcohol.

## Electronic supplementary material

Supplementary Information
