# Supplementary material for: Genotype determination of the OPN1LW/OPN1MW genes: novel disease-causing mechanisms in Japanese patients with blue cone monochromacy
Source: Sci Rep. 2018 Jul 31;8:11507. doi: 10.1038/s41598-018-29891-9 (PMC6068165; doi:10.1038/s41598-018-29891-9)
Supplement: Supplementary file 1 — Supplementary Information [file 41598_2018_29891_MOESM1_ESM.pdf]

Supplementary Information

Title

Genotype determination of the *OPN1LW*/*OPN1MW* genes: novel disease-causing mechanisms in Japanese patients with blue cone monochromacy

Authors

Satoshi Katagiri, MD, PhD[1*], Maki Iwasa, MD[2*], Takaaki Hayashi, MD, PhD[1,3], Katsuhiro Hosono, PhD[4], Takahiro Yamashita, PhD[5], Kazuki Kuniyoshi, MD, PhD[6], Shinji Ueno, MD, PhD[7], Mineo Kondo, MD, PhD[8], Hisao Ueyama, MD, PhD[9], Hisakazu Ogita, MD, PhD[9], Yoshinori Shichida, PhD[5], Hidehito Inagaki, PhD[10], Hiroki Kurahashi, MD, PhD[10], Hiroyuki Kondo, MD, PhD[11], Masahito Ohji, MD, PhD[2], Yoshihiro Hotta MD, PhD[4], Tadashi Nakano, MD, PhD[1]

*These authors equally contributed to this work.

1 Department of Ophthalmology, The Jikei University School of Medicine, Tokyo, Japan
2 Department of Ophthalmology, Shiga University of Medical Science, Shiga, Japan
3 Department of Ophthalmology, Katsushika Medical Center, The Jikei University School of Medicine, Tokyo, Japan
4 Department of Ophthalmology, Hamamatsu University School of Medicine, Shizuoka, Japan
5 Department of Biophysics, Graduate School of Science, Kyoto University, Kyoto, Japan.
6 Department of Ophthalmology, Kindai University Faculty of Medicine, Osaka, Japan
7 Department of Ophthalmology, Nagoya University Graduate School of Medicine, Aichi, Japan
8 Department of Ophthalmology, Mie University Graduate School of Medicine, Mie, Japan
9 Department of Biochemistry and Molecular Biology, Shiga University of Medical Science, Shiga, Japan
10 Division of Molecular Genetics, Institute for Comprehensive Medical Science,
Fujita Health University, Aichi, Japan
11 Department of Ophthalmology, University of Occupational and Environmental Health, Fukuoka, Japan.

Supplemental Table S1. Primers used in this study

	Primer name	Sequence (5' to 3')	Position*	Position (NT_025965.12)**
For long-range PCR of first and downstream genes	FG	AAGCCAACAGCAGGATGTGCG	–565 to –545 (first gene)	761,115 to 761,135
	DG	AAAGCCTAACAATGTCCAGGG	–748 to –728 (downstream gene)	799,341 to 799,361
	E6R	GCAGTGAAAGCCTCTGTGACTT	exon 6	776473 to 776494, and 813,603 to 813,624
For sequencing of promoter + exon 1 (and adjacent intron 1)	FGP	GGCGGACGCAGGACAGTAGAA	–309 to –289 (first gene)	761,371 to 761,391
	DGP	AGACAGAGTCTTGGTCTGTTG	–317 to –297 (downstream gene)	799,772 to 799,792
For sequencing of exons 2-6	E2F	AGGATGGACAAAGCTGGAGG	intron 1	767,951 to 767,970, and 805,082 to 805,101
	E3F	TGGTGGAAAGAAAGATGTCG	intron 2	770,225 to 770,244, and 807,355 to 807,374
	E4F	CAAATTGGGTAATCTCATGC	intron 3	771,872 to 771,891, and 809,002 to 809,021
	E5F	GCTGTGCTCCACTCAGGGCT	intron 4	773,604 to 773,623, and 810,734 to 810,753
	E6F	GAAATAATCCAAGCCTTCCT	intron 5	776,125 to 776,144, and 813,255 to 813,274
For PCR of promoter	PROMF	GAGGAGGAGGTCTAAGTCCC	–128 to –109 (first and downstream genes)	761,552 to 761,571, and 799,961 to 799,980
	PROMR	GGCTATGGAAAGCCCTGTCCC	+21 to +41 (Exon 1 of L/M genes)	761,700 to761,720, and 800,109 to 800,129
For PCR of locus control region	LCRF	AAGTGTCAAAGGCAAATGGC	upstream of LCR	757,639 to 757,658
	LCRR	ATCCAAGAATGTGAGACC	downstream of LCR	758,690 to 758,707
For PCR and sequencing of deleted region (Case 3)	UP8F	AATGTGGTCCATCTCGGTGAA	–32,330 to –32,310 (first gene)	729,350 to 729,370
	UP8R	TCAATTCTCCAAGAATACACA	–32,036 to –32,016 (first gene)	729,644 to 729,664
	E5R	TTCTTATCAGAGACATGATT	intron 5	774,046 to 774,065, and 811,176 to 811,195
	UP9F	GTCTACCCCCTGAGTTTGGCG	–28,506 to –28,486 (first gene)	733,174 to 733,194
	UP9R	AGGACAGTCTCTCCAGCCAGA	–28,170 to –28,150 (first gene)	733,510 to 733,530
	UP12F	TCTACAGGTGTCTACATGTC	–31,565 to –31,544 (first gene)	730,115 to 730,134
For deleted region (Case 4)	I1R1	GGGGGCAAAGGAGAGAACAGG	intron 1	764,660 to 764,680, and 803,071 to 803,091
	I1R2	CTTGAGGGGAAGACCACTA	intron 1	767,643 to 767,661, and 804,774 to 804,792
	IGR1	CCCTCAGACGCCTACTTTATG	intergenic region	791,231 to 791,251
	IGR2	AGTTTTGCCTGCTCTTGGAAT	intergenic region	795,380 to 795,400
	I1F	GAGAGCTAGAGAGCACTCT	intron 1	767,165 to 767,183, and 804,296 to 804,314

PCR = polymerase chain reaction, *The number is from the cap site of each gene., **The positions in the downstream gene are those of *OPN1MW1*.

Supplemental Table S2. Clinical findings of four cases with blue cone monochormacy

Case	Patient ID	Age at examination	Gender	Inheritance	BCVA	Refraction	Funduscopy	Farnsworth panel D-15	Full-field ERG	S-cone ERG	Photophobia	Nystagmus	Progression
1	JU#1299	8 years	M	X-linked	RE: 0.15<br>LE: 0.15	RE: -1.50 D<br>LE: -1.75D	BE: Normal appearance	Protan-like pattern	Rod response: normal<br>Cone-rod response: normal<br>Cone response: absent<br>30-Hz flicker: absent	Detected	Yes	Yes	No
2	JU#1311 (KINKI-125-70)	6 years	M	X-linked	RE: 0.1<br>LE: 0.15	RE: +1.50 D<br>LE: +1.50 D	BE: Normal appearance	Protan pattern	Rod response: normal<br>Cone-rod response: normal<br>Cone response: absent<br>30-Hz flicker: absent	Detected	Yes	Yes	No
3	JU#1318 (MIE-050-0071)	19 years	M	X-linked	RE: 0.3<br>LE: 0.3	RE: -6.75 D<br>LE: -7.75D	BE: Normal appearance	Protan-like pattern	Rod response: normal<br>Cone-rod response: normal<br>Cone response: absent<br>30-Hz flicker: absent	Detected	No	Yes	No
4	#1368 (Nagoya-140)	66 years	M	X-linked	RE: 0.25<br>LE: 0.13	RE:psudophakia<br>LE:psudophakia	BE: Chorioretinal atropy including macular degeneration and peripapillary atrophy	Protan pattern	Rod response: decreased<br>Cone-rod response: decreased<br>Cone response: absent<br>30-Hz flicker: absent	Detected	No	No	Progressive myopia

M = male, BCVA = best corrected visual acuity, RE = right eye, LE = left eye, BE = both eyes, D = diopter, ERG= electroretinography

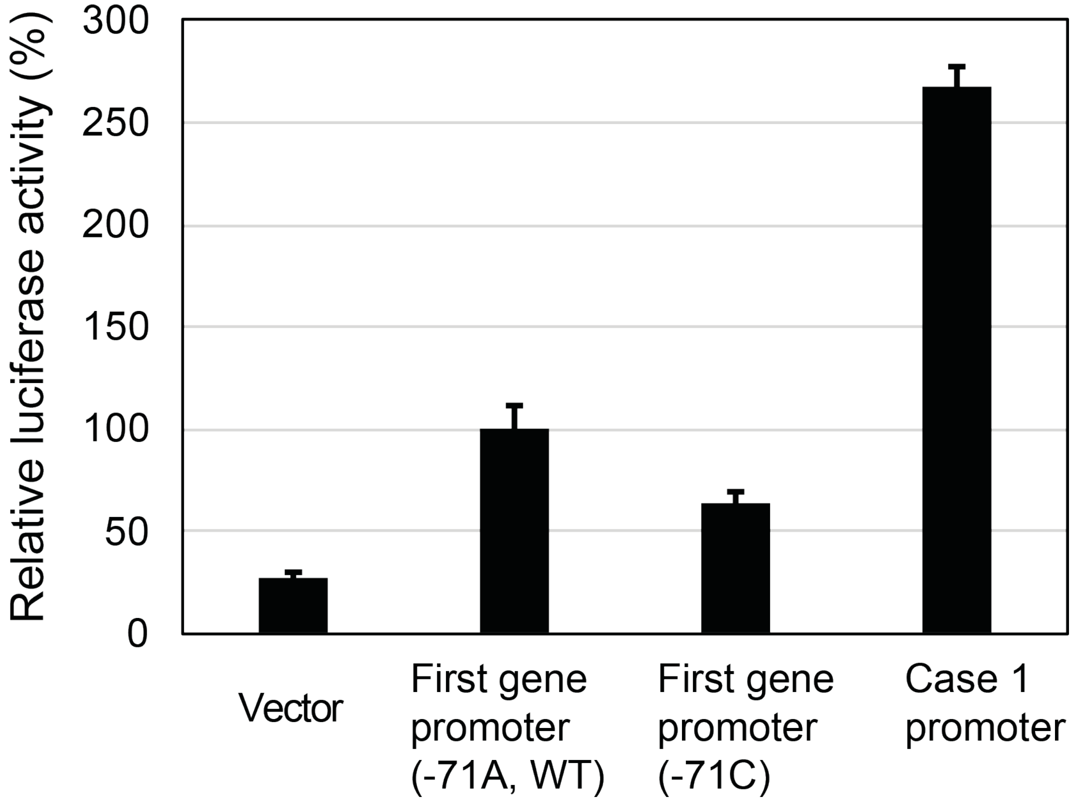

Supplemental Figure S1. Results of promoter assay using a luciferase reporter vector

The promoter region was cloned in a luciferase reporter vector and transfected into WERI cells. The luciferase activities of three independent experiments each, shown as an average with its standard deviation, are expressed as relative values, with the activity of wild type (WT) first gene promoter being 100%.

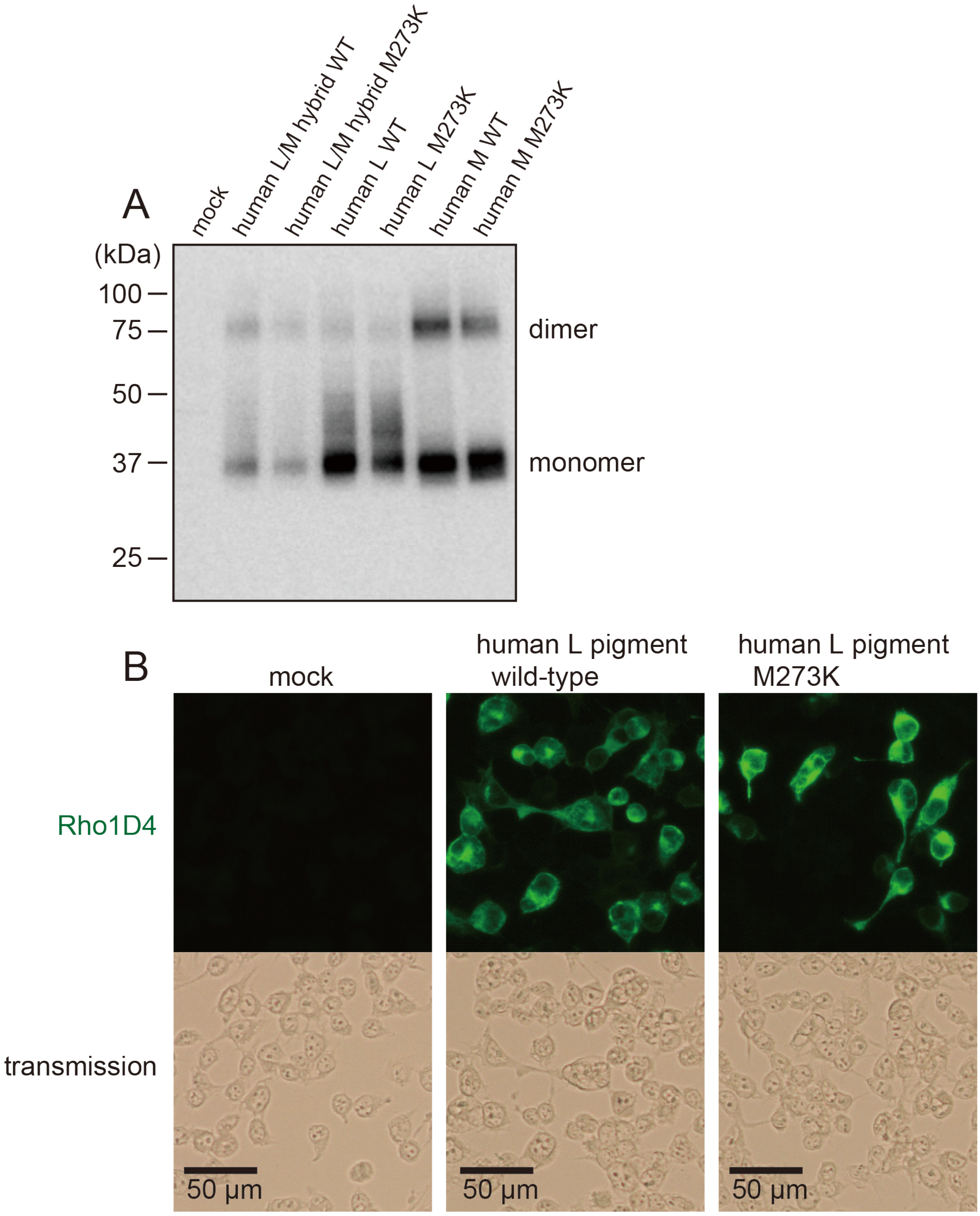

Supplemental Figure S2. Analysis of M273K mutant pigments

(A) Western blot analysis of M273K mutants of human L, M and L/M hybrid pigments. Rho1D4 monoclonal antibody detects the C-terminal tag of the pigments in the extracts of pigment-expressed HEK293 cells. The spectral analysis shows that the expression level of the recombinant proteins of L/M hybrid pigment is about 10-times lower than that of M pigment (Fig. 3C), which is consistent with the results of the western blot analysis. The apoproteins of M273K mutant pigments are significantly detected in the extracts of the transfected cells. (B) Immunohistochemical analysis of human L pigment M273K mutant in HEK293 cells. Rho1D4-labeled fluorescence images and transmission images of human L pigment wild-type or M273K mutant-expressed HEK293 cells are shown. The subcellular localization of M273K mutant is quite similar to that of wild-type human L pigment. A control of mock-transfected cells shows no fluorescence. Thus, the disfunction of M273K mutant pigments (Fig. 3C) is probably due to a deficient incorporation of 11-cis-retinal, not due to misfolding or mistrafficking in the cells.

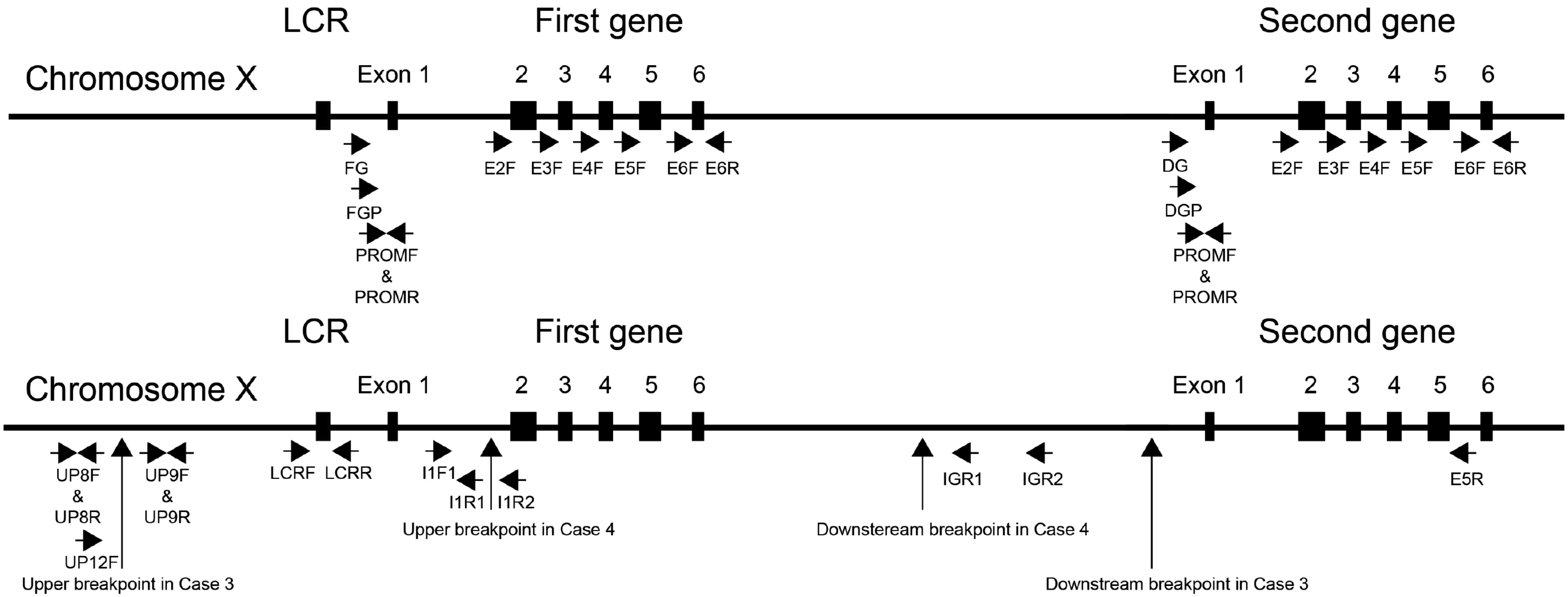

Supplemental Figure S3. Overview of primer position

The position of primers used in this study are shown in the general composition of an L/M gene array (locus control region and first and second genes).
